# Genotoxicity Evaluation of Metformin in Freshwater Planarian *Dugesia japonica* by the Comet Assay and RAPD Analysis

**DOI:** 10.1155/2022/2822605

**Published:** 2022-08-17

**Authors:** Dandan Yin, Zhenbiao Xu, Minmin Feng, Zelong Zhao, Dahu Chen, Linxia Song

**Affiliations:** School of Life Sciences and Medicine, Shandong University of Technology, Zibo 255049, China

## Abstract

**Objective:**

To investigate the genotoxicity of metformin on planarian with different concentrations and exposure times.

**Methods:**

The planarians were treated, respectively, with 10 mmol/L and 50 mmol/L metformin for 1, 3, and 5 days, and then, the comet assay and random amplified polymorphic DNA (RAPD) analysis were performed. 13 random primers were used for PCR amplification with the genomic DNAs as templates. Planarians cultured in clear water were used as the control. Genomic template stability (GTS) was calculated by comparing and analyzing the RAPD patterns of the control group and the treatment groups.

**Results:**

In the comet assay, DNA damage of planarians treated with 10 mmol/L metformin for 1, 3, and 5 days was 10.2%, 25.4%, and 36.8%, respectively, and that of planarians treated with 50 mmol/L metformin was 40.6%, 62.8%, and 65.4%, respectively. GTS values of planarians exposed to 10 mmol/L metformin for 1, 3, and 5 days were 64.1%, 62.8%, and 52.6%, respectively, and those of planarians exposed to 50 mmol/L metformin for 1, 3, and 5 days were 52.6%, 51.3%, and 50%, respectively. DNA damage increased and GTS values decreased with the increasing metformin exposure concentration and exposure time.

**Conclusion:**

Metformin has certain genotoxicity on planarian in a dose- and time-related manner. The comet assay and RAPD analysis are highly sensitive methods for detecting genotoxicity with drugs.

## 1. Introduction

Metformin is a first-line drug for the treatment of type II diabetes [1]. It is widely used all over the world because of its efficacy, low cost, weight neutrality, and good safety profile [2]. However, metformin has become one of the most common pharmaceuticals found in wastewater and river basins all over the world with the increasing prescription and consumption [3]. Studies showed that metformin has some certain toxicity to aquatic organisms. Siamese fighting fish (*Betta splendens*) exposed to metformin at environmental concentration exhibited less aggressive behaviors toward the male conspecific stimulus with substantial reductions in gill flaring and fin spreading, and these behaviors are very important for their territorial defense and mate acquisition [4]. Exposing fathead minnows (*Pimephales promelas*) to metformin with a concentration equivalent to that in wastewater led to the development of intersex gonads in males and reduced the size of male fish and the fertility of treated pairs [5]. Metformin induced several morphological alterations such as malformation of a tail, scoliosis, pericardial edema, and yolk deformation on zebrafish embryos [6]. Japanese medaka could significantly reduce growth metrics, alter metabolomes, and change the expression of genes associated with cell growth in the early life stage when exposed to a range of relevant concentrations of metformin [7]. So, we hypothesize that metformin also affects the behavior of freshwater planarian and further causes damage to its genome. But until now, there are few reports on the genetic toxicity of metformin to organisms detected by the comet assay and random amplified polymorphic DNA (RAPD) technology.

The comet assay is a well-established, simple, reliable, and sensitive method for the detection of DNA damage in individual cells [8]. This method was widely used in genetic toxicology, human biomonitoring, medical diagnosis, and ecological assessment of sentinel organisms exposed to environmental contaminants [9–12]. RAPD is a technique for detecting polymorphisms at the molecular level of DNA, which was often used to detect the effects of metal elements, chemicals, and drugs on the genetic material of animals and plants [13–15]. It was also used to determine the genotoxicity of environmental pollutants and some carcinogens in aquatic animals [16–18].

Planarians are metazoan flatworms, belonging to the phylum *Platyhelminthes* and class *Turbellaria*. Planarians are very sensitive to toxic substances in the environment; sublethal doses of pesticides and heavy metals could cause morphological, histological, and DNA changes to them [19, 20]. Due to their strong regenerative ability and high chemical sensitivity, planarians have become model organisms for regenerative medicine, stem cells, neurological diseases, and toxicology research [21, 22]. The purpose of this work is to investigate the genotoxicity of metformin in planarian by the comet assay and RAPD analysis, so as to evaluate the potential genetic impact of metformin on aquatic organisms and lay a good foundation for further study of the toxicological mechanism of metformin.

## 2. Materials and Methods

### 2.1. Test Animals

Planarians (*D. japonica*) used in this work are the asexual strain *Dugesia ZB-1* [23], which are from Zibo, Shandong Province, China, cultured in commercially mineral water or Montjuïc water (1.6 mmol/L NaCl, 1.0 mmol/L CaCl_2_, 1.0 mmol/L MgSO_4_, 0.1 mmol/L MgCl_2_, 0.1 mmol/L KCl, and 1.2 mmol/L NaHCO_3_), at 20-24°C in a biochemical incubator (SPX-2508SH, Shanghai CIMO Medical Instrument Manufacturing Co., Ltd., China). Animals are fed with beef liver twice a week and starved for a week before the experiment. Animal experiments were conducted under the supervision and assessment by the Laboratory Animal Ethics Committee of Shandong University of Technology.

### 2.2. Materials

Metformin was purchased from Adamas-Beta, Inc. Trypsin, low melting point agarose, and DAPI were purchased from Solarbio Company. CometSlide was the product of Trevigen Company. Nucleic acid dye RbGreen (RB109-500) was purchased from Beijing Ruibo Xingke Biotechnology Co., Ltd. The E.Z.N.A.® Mollusc DNA Kit (D3373-01) was purchased from Omega Bio-Tek Company, and 2×Taq PCR StarMix was from GenStar Company. 1 kb DNA ladder (N3232L) was from New England Biolabs Company, and DL2000 DNA marker (3427A) was from TaKaRa Company. The sequences of 13 random primers used in this study are shown in Table S1 in Supplementary Materials.

### 2.3. Monitoring of Morphological Changes and Mortality

Two groups of planarians were treated in Petri dishes with 10 mmol/L and 50 mmol/L metformin for 5 days. The control planarians were cultured in clean water. Each group contained 10 planarians, and the experiment was repeated three times. The treatment solutions were changed every day, and the morphological changes and mortality of planarians were observed. Morphological images were obtained by NIS-Elements F software.

### 2.4. Comet Assay

Planarians treated with 10 mmol/L and 50 mmol/L metformin for 1, 3, and 5 days were washed with 1× PBS buffer and then digested with 1% trypsin at room temperature for about 20 min until tissues were completely dissolved. Samples were centrifuged at 2000 rpm for 5 min at 4°C to obtain cell precipitation, and then, 1 mL 1× PBS was added to resuspend the cells. The cell suspension was filtered with a 70 *μ*m cell strainer and centrifuged at 2000 rpm for 5 min at 4°C. Resuspend the cell precipitation with 20 *μ*L 1× PBS to obtain single-cell suspension. Mix the cell suspension with 1% low melting agarose at a ratio of 1 : 5 (*v*/*v*), and place 50 *μ*L mixture on the CometSlide. Place the slide at 4°C for 30 min, and then, immerse it into a precooled cell lysing solution (2.5 mol/L NaCl, 100 mmol/L Na_2_EDTA, 10 mmol/L Tris, 1% sodium sarcosinate, 1% Triton X-100, and 10% DMSO) at 4°C for 1 h. The slide was washed three times with 1× PBS and then placed into the freshly prepared cold alkaline buffer (1 mmol/L Na_2_EDTA and 30 mmol/L NaOH, pH > 13) at 4°C for 30 min. Electrophoresis was performed at 25 V and 200 mA for 20 min in alkaline buffer. After electrophoresis, cells were stained with 2 *μ*g/mL DAPI and observed under a fluorescence microscope (Olympus DP80, Japan). 50 cells were randomly selected from each slide, and the percentage of tail DNA and Olive Tail Moment (OTM) value were analyzed with CASP software.

### 2.5. RAPD Procedures

#### 2.5.1. Genomic DNA Extraction

Planarians were treated with metformin at concentrations of 10 mmol/L and 50 mmol/L for 1, 3, and 5 days at constant temperature at 20°C, and planarians cultured in clear water were used as the control. The genomic DNAs of planarians were extracted according to the instructions of the DNA kit. 2 *μ*L stained DNA with RbGreen was checked by electrophoresis on 0.8% agarose gels in 1× TAE buffer to detect the integrity. DNA images were obtained with AlphaView software. The purity of DNA was measured by detecting OD_260_/OD_280_ with a micro-spectrophotometer (K5600, Beijing Kaiao Technology Development Co., Ltd., China).

#### 2.5.2. RAPD-PCR

Each PCR reaction was performed in a mixture of 25 *μ*L containing 20 ng of genomic DNA, 0.2 *μ*mol/L primer, and 12.5 *μ*L 2× Taq PCR StarMix. Amplification was implemented in a gene amplification device (TC-XP, Hangzhou Bioer Technology Co., Ltd., China) programmed for 5 min at 94°C and 40 continuous cycles each consisting of 1 min at 94°C, 1 min at 37°C, and 2 min at 72°C, followed by 10 min at 72°C with a final extension. After amplification, the PCR products were analyzed by electrophoresis on 1% agarose gels in 1× TAE buffer. The electropherograms were photographed under an AlphaImager HP system (Alpha2200-5, Alpha Innotech, USA).

#### 2.5.3. Estimate of Genomic Template Stability (GTS)

GTS is the percentage of the number of polymorphic bands to the total number of bands of the control group in the RAPD maps. GTS was calculated according to the formula: GTS (%) = (1 − *a*/*n*) × 100. “*a*” is the number of polymorphic bands detected in each treatment group, and “*n*” is the total number of bands in the control group. Polymorphisms in the RAPD profile include disappearance of normal bands and appearance of new bands in the treatment groups compared with the control group. To facilitate comparison, the GTS of the control group is set to 100%, and the GTS of each treatment group is expressed as a percentage of the control group.

### 2.6. Data Analysis

Statistical analysis was performed using SPSS 24.0 software. The percentage of tail DNA and OTM values was presented as the mean ± SD. One-way ANOVA was used to compare means among groups; a value of *p* < 0.05 was considered to be statistically significant.

## 3. Results

### 3.1. Morphological Changes after Treatment with Metformin

**Figure 1:**
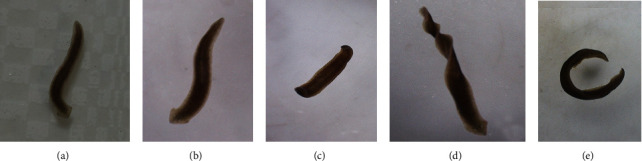
Morphological changes of *D. japonica* treated with metformin at different concentrations. The control group of planarians (a) and those exposed to 10 mmol/L metformin (b) have normal morphology. Planarians exposed to 50 mmol/L metformin appeared to exhibit bridge-like position (c), screw-like hyperkinesia (d), and C-like position (e).

Planarians in the control group and 10 mmol/L metformin-treated group showed no morphological changes during the experiment; they exhibited normal morphology and sliding behavior. However, planarians treated with 50 mmol/L metformin showed varying degrees of body curling, forming bridge-like position, screw-like hyperkinesia, and C-like position (Figure 1). No death occurred during the treatment time.

### 3.2. DNA Damage Detected by the Comet Assay

**Figure 2:**
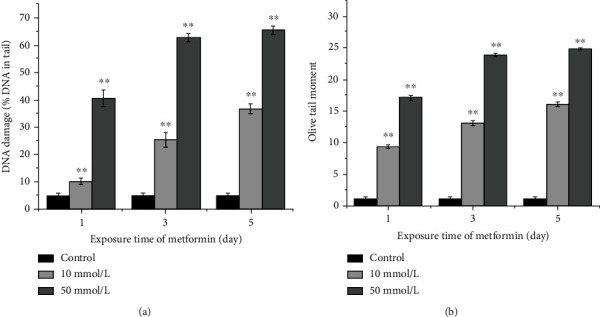
DNA damage represented by the percentage of DNA in the tail of the comet and OTM value in *D. japonica* cells after different concentrations and exposure times treated with metformin: (a) DNA damage by the percentage of DNA in the tail; (b) OTM value. ∗ means significant difference (*p* < 0.05); ∗∗ means extremely significant difference from the control (*p* < 0.01).

The effects of metformin on DNA damage in the planarians were evaluated by the comet assay, and the results are shown in Figure 2. Compared with the control, planarians treated with metformin showed obvious DNA damage. Analysis of the percentage of tail DNA showed that DNA damage of planarians treated with 10 mmol/L metformin for 1, 3, and 5 days was 10.2%, 25.4%, and 36.8%, respectively, and that of planarians treated with 50 mmol/L was 40.6%, 62.8%, and 65.4%, respectively. Analysis of OTM values showed that the values in 10 mmol/L metformin-treated groups for 1, 3, and 5 days were 9.4%, 13.1%, and 16.1%, respectively, and those in groups treated with 50 mmol/L were 17.1%, 23.9%, and 24.9%, respectively. The percentage of tail DNA and the OTM values gradually increased with the increase in the metformin concentration and the exposure time, indicating that the extent of DNA damage has a certain positive correlation with the dose and the exposure time of metformin.

### 3.3. Effect of Metformin on the GTS of Planarian by RAPD Analysis

**Figure 3:**
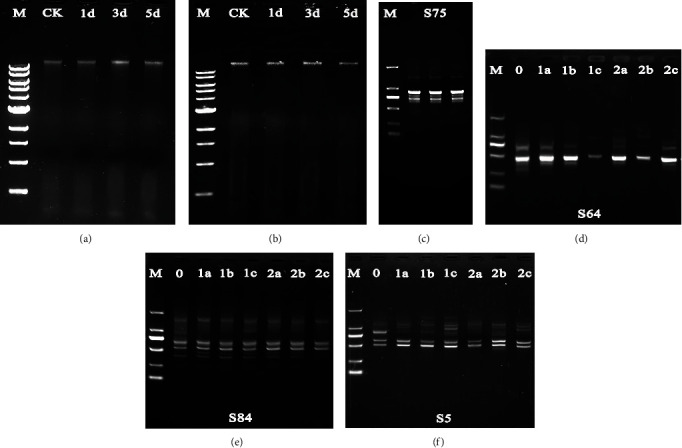
Genomic DNAs and RAPD profiles of planarian *D. japonica*. (a, b) The quality of genomic DNAs isolated from *D. japonica* treated with 10 mmol/L (a) and 50 mmol/L (b) metformin for 1, 3, and 5 days. M is the 1 kb DNA ladder (10,000, 8000, 6000, 5000, 4000, 3000, 2000, 1500, 1000, and 500 bp from top to bottom). CK is control. (c) Reproducibility of RAPD profiles generated from planarian genomic DNA by primer S75. M is the DL2000 DNA marker (2000, 1000, 750, 500, 250, and 100 bp from top to bottom). (d–f) RAPD profiles of genomic DNAs from *D. japonica* treated with metformin using primers S64, S84, and S5. 0 is control; 1 and 2 represent 10 mmol/L and 50 mmol/L; a, b, and c represent 1, 3, and 5 days, respectively; M is the DL2000 DNA marker.

The purity of the genomic DNAs was determined spectrophotometrically as 1.7~2.2, indicating a high degree of DNA purity. The isolated DNAs appeared to be a single band (Figures 3(a) and 3(b)), indicating that the DNA integrity was good and there was no degradation. The amplification with the same primer always appeared to have the same bands (Figure 3(c)), indicating that the RAPD assay was repeatable.

Compared with the untreated control, differences in RAPD patterns of the treatment groups were the gain or loss of the DNA band and the variation of band intensity (Figures 3(d)–3(f)). The number of polymorphic bands in 10 mmol/L and 50 mmol/L treatment groups was 28, 29, and 37 and 37, 38, and 39 after 1, 3, and 5 days of exposure, respectively (Table S2 in Supplementary Materials). The number of varied bands in 10 mmol/L and 50 mmol/L treatment groups was 52, 56, and 69 and 64, 66, and 70 for 1, 3, and 5 days of exposure, respectively. These results indicate that the RAPD pattern of metformin-treated groups was different from that of the control group and exhibited obvious changes with the increase in metformin concentration and exposure time.

**Figure 4:**
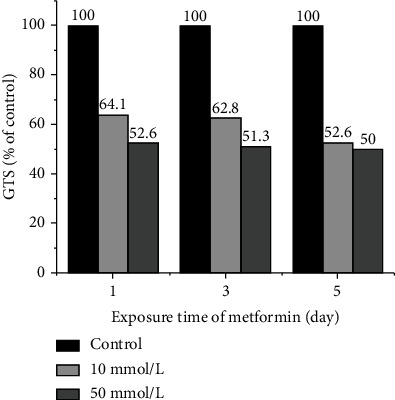
Genomic template stability (GTS) of *D. japonica* treated with 10 mmol/L and 50 mmol/L metformin for 1, 3, and 5 days.

The GTS values of the treatment groups were calculated based on the RAPD patterns amplified from the 13 primers. Compared with the control group, the GTS values of 10 mmol/L and 50 mmol/L treatment groups were 64.1%, 62.8%, and 52.6% and 52.6%, 51.3%, and 50% after exposure for 1, 3, and 5 days, respectively (Figure 4). At the same exposure time, the GTS values decreased with the increase in metformin concentration, while at the same metformin concentration, the GTS values decreased with the extension of exposure time, indicating that metformin has genotoxic effect on planarian in a dose- and time-related manner.

## 4. Discussion

In the present days, the increasing number of genotoxic pollutants in the aquatic environment has attracted widespread attention and led to the development of rapid monitoring methods. The comet assay and RAPD technology have been widely used in the field of ecotoxicology and biomonitoring because of their simplicity, rapidity, and sensitivity. Among the traditional environmental indicators, planarian has become a more suitable bioindicator in some respects due to its high sensitivity, low cost, high proliferation, and regeneration rate [24]. Planarians are widely distributed in the world, and three planarian species *Dugesia japonica*, *Schmidtea mediterranea*, and *Girardia tigrina* are commonly used in toxicology research [25]. The planarians used in this experiment are an asexual strain *Dugesia ZB-1* established by Xu et al. [23]. Because the planarians have the same genetic background, the difference of genetic polymorphism among individuals was reduced, which made our experimental data more stable and accurate.

Nowadays, with the increasing prevalence of diabetes, metformin has become one of the most commonly prescribed drugs in the world. Due to the incomplete metabolism of metformin in human body, about 70% of the therapeutic dosage is excreted through urine and feces, resulting in its extensive exposure in the environment [7, 26]. The increasing metformin detected in aquatic environment has raised concerns about its potential ecotoxicological effects. In this study, the genotoxicity of metformin exposure to freshwater planarian was investigated by the comet assay and RAPD technology. The genotoxicity induced by different concentrations of metformin (10 mmol/L and 50 mmol/L) at different times (1, 3, and 5 days) was analyzed. The results showed that with the increase in metformin concentration and exposure time, DNA damage represented by the percentage of tail DNA and OTM gradually increased, and GTS gradually decreased, indicating that the genotoxic effect of metformin on planarians was dose- and time-related. From the result of morphological observation, 10 mmol/L metformin did not cause morphological changes of planarians. However, through the detection of DNA damage and its polymorphism, it was found that metformin at this concentration had caused certain damage to the DNA of planarians, and the DNA damage gradually increased with the extension of exposure time, indicating that the comet assay and RAPD technology are more sensitive methods than simple morphological observation for detecting the toxicity of drugs.

In addition to genotoxicity, metformin also has other effects on aquatic organisms. It affected the endocrine function of juvenile fathead minnows *Pimephales promelas* (FHM) by upregulating the expression of estrogen-associated genes VTG, ER*α*, GnRH3, and CYP3A4 [27]. Low-dose metformin exposure for a long time could cause a significant upregulation of numerous endocrine-related genes in FHM [28]. Metformin affected the expression of genes related to neurological and cardiovascular development and the expression of CYP3A65, GSTM1, p53, and DNMT1 genes in the liver in zebrafish [29, 30]. It was also found that metformin could induce oxidative stress and the two-generation endocrine disruption in *Oryzias latipes* [31]. All the above studies showed that metformin is toxic to organisms through a variety of ways. Therefore, it remains an important area of investigation to analyze physiological and biochemical parameters in combination with gene expression and signal transduction to clarify the mechanism of metformin toxicity on planarian in the future.

## 5. Conclusion

The results of the present study revealed that metformin can cause genotoxicity on planarian; the genotoxicity is positively correlated with the concentration and the exposure time of metformin. This study also indicates that the comet assay and RAPD are highly sensitive methods for detecting genotoxicity induced by drugs in aquatic environment.

## Data Availability

The data used to support the findings of this study are included within the article and can be available from the corresponding author upon request.

## Supplementary Materials

Supplementary 1 Table S1: sequences of 13 primers used in this experiment.

Supplementary 2 Table S2: changes of total bands in control and of polymorphic bands and varied bands in metformin-treated *D. japonica*.
