# Supplementary material for: Genotoxicity Evaluation of Metformin in Freshwater Planarian Dugesia japonica by the Comet Assay and RAPD Analysis
Source: Biomed Res Int. 2022 Aug 17;2022:2822605. doi: 10.1155/2022/2822605 (PMC9403254; doi:10.1155/2022/2822605)
Supplement: Supplementary 1 — Table S1: sequences of 13 primers used in this experiment. [file 2822605.f1.docx]

**Table S1**

Sequences of 13 primers used in this experiment.

| Primer | Sequence（5′ ⟶ 3′） | GC content |
| --- | --- | --- |
| S5 | TGCGCCCTTC | 70% |
| S8 | GTCCACACGG | 70% |
| S10 | CTGCTGGGAC | 70% |
| S15 | GGAGGGTGTT | 60% |
| S17 | AGGGAACGAG | 60% |
| S18 | CCACAGCAGT | 60% |
| S20 | GGACCCTTAC | 60% |
| S64 | CCGCATCTAC | 60% |
| S75 | GACGGATCAG | 60% |
| S78 | TGAGTGGGTG | 60% |
| S80 | ACTTCGCCAC | 60% |
| S83 | GAGCCCTCCA | 70% |
| S84 | AGCGTGTCTG | 60% |
