# Supplementary material for: Genotoxicity Evaluation of Metformin in Freshwater Planarian Dugesia japonica by the Comet Assay and RAPD Analysis
Source: Biomed Res Int. 2022 Aug 17;2022:2822605. doi: 10.1155/2022/2822605 (PMC9403254; doi:10.1155/2022/2822605)
Supplement: Supplementary 2 — Table S2: changes of total bands in control and of polymorphic bands and varied bands in metformin-treated D. japonica. [file 2822605.f2.docx]

**Table S2**

Changes of total bands in control and of polymorphic bands and varied bands in metformin treated *D. japonica*.

| **Primers** | **Control** | **Metformin(mmol/L)** | | | | | | | | | | | | | | | | | | | | | | | | | | | | |
| --- | --- | --- | --- | --- | --- | --- | --- | --- | --- | --- | --- | --- | --- | --- | --- | --- | --- | --- | --- | --- | --- | --- | --- | --- | --- | --- | --- | --- | --- | --- |
|  |  | **10** | | | | | | | | | | | | | |  | **50** | | | | | | | | | | | | | |
|  |  | **1d** | | | |  | **3d** | | | |  | **5d** | | | |  | **1d** | | | |  | **3d** | | | |  | **5d** | | | |
|  |  | **a** | **b** | **c** | **d** |  | **a** | **b** | **c** | **d** |  | **a** | **b** | **c** | **d** |  | **a** | **b** | **c** | **d** |  | **a** | **b** | **c** | **d** |  | **a** | **b** | **c** | **d** |
| S5 | 5 | 1 | 1 | 3 | 0 |  | 1 | 0 | 2 | 2 |  | 2 | 0 | 3 | 1 |  | 1 | 1 | 1 | 1 |  | 2 | 0 | 3 | 1 |  | 1 | 1 | 4 | 0 |
| S8 | 11 | 1 | 3 | 4 | 0 |  | 1 | 4 | 4 | 1 |  | 2 | 5 | 4 | 1 |  | 0 | 6 | 3 | 0 |  | 3 | 3 | 2 | 0 |  | 2 | 5 | 3 | 1 |
| S10 | 5 | 2 | 1 | 0 | 3 |  | 0 | 1 | 0 | 3 |  | 2 | 1 | 0 | 2 |  | 2 | 0 | 0 | 4 |  | 0 | 0 | 0 | 4 |  | 3 | 0 | 0 | 3 |
| S15 | 3 | 0 | 0 | 1 | 0 |  | 0 | 0 | 1 | 1 |  | 0 | 0 | 1 | 0 |  | 0 | 0 | 1 | 1 |  | 0 | 0 | 2 | 0 |  | 0 | 0 | 0 | 1 |
| S17 | 10 | 0 | 6 | 2 | 1 |  | 1 | 6 | 1 | 0 |  | 0 | 2 | 5 | 0 |  | 2 | 4 | 4 | 0 |  | 2 | 5 | 4 | 0 |  | 2 | 2 | 4 | 0 |
| S18 | 10 | 0 | 2 | 3 | 0 |  | 0 | 0 | 4 | 0 |  | 0 | 2 | 3 | 1 |  | 1 | 1 | 2 | 1 |  | 1 | 3 | 2 | 0 |  | 1 | 3 | 4 | 0 |
| S20 | 3 | 1 | 0 | 0 | 0 |  | 1 | 0 | 0 | 0 |  | 1 | 0 | 0 | 1 |  | 1 | 2 | 0 | 0 |  | 0 | 1 | 0 | 1 |  | 2 | 0 | 0 | 3 |
| S64 | 4 | 0 | 1 | 1 | 1 |  | 0 | 1 | 0 | 1 |  | 1 | 3 | 0 | 1 |  | 2 | 1 | 0 | 1 |  | 2 | 3 | 0 | 1 |  | 1 | 0 | 0 | 2 |
| S75 | 5 | 1 | 0 | 1 | 1 |  | 2 | 1 | 0 | 0 |  | 2 | 0 | 0 | 0 |  | 2 | 2 | 0 | 0 |  | 3 | 0 | 0 | 0 |  | 2 | 2 | 0 | 0 |
| S78 | 4 | 1 | 0 | 0 | 0 |  | 1 | 0 | 1 | 0 |  | 1 | 0 | 2 | 0 |  | 1 | 0 | 2 | 0 |  | 0 | 0 | 0 | 1 |  | 1 | 1 | 0 | 0 |
| S80 | 8 | 1 | 2 | 0 | 2 |  | 4 | 2 | 0 | 3 |  | 5 | 2 | 2 | 3 |  | 2 | 1 | 1 | 2 |  | 2 | 3 | 2 | 1 |  | 4 | 2 | 2 | 2 |
| S83 | 4 | 0 | 0 | 0 | 0 |  | 0 | 0 | 1 | 0 |  | 0 | 1 | 0 | 1 |  | 1 | 0 | 2 | 0 |  | 0 | 1 | 2 | 0 |  | 0 | 1 | 0 | 0 |
| S84 | 6 | 2 | 2 | 1 | 0 |  | 2 | 2 | 1 | 1 |  | 3 | 2 | 0 | 1 |  | 2 | 2 | 1 | 0 |  | 2 | 2 | 1 | 1 |  | 1 | 2 | 0 | 2 |
| Total bands | 78 | 10 | 18 | 16 | 8 |  | 13 | 17 | 15 | 12 |  | 19 | 18 | 20 | 12 |  | 17 | 20 | 17 | 10 |  | 17 | 21 | 18 | 10 |  | 20 | 19 | 17 | 14 |
| a+b |  | 28 |  |  |  |  | 29 |  |  |  |  | 37 |  |  |  |  | 37 |  |  |  |  | 38 |  |  |  |  | 39 |  |  |  |
| a+b+c+d |  | 52 |  |  |  |  | 56 |  |  |  |  | 69 |  |  |  |  | 64 |  |  |  |  | 66 |  |  |  |  | 70 |  |  |  |

a: appearance of new bands, b: disappearance of normal bands, c: increase in band intensities, d: decrease in band intensities, a+b: polymorphic bands, a+b+c+d: varied bands.
